# Novel high-coverage targeted metabolomics method (SWATHtoMRM) for exploring follicular fluid metabolome alterations in women with recurrent spontaneous abortion undergoing in vitro fertilization

**DOI:** 10.1038/s41598-019-47370-7

**Published:** 2019-07-26

**Authors:** Jingyan Song, Xiaoming Wang, Ying Guo, Yi Yang, Kaiyue Xu, Tianqi Wang, Yuanhong Sa, Lihua Yuan, Huaying Jiang, Jiayin Guo, Zhengao Sun

**Affiliations:** 1 Reproductive and Genetic Center of Integrated Traditional and Western Medicine, The Affiliated Hospital of Shandong University of Traditional Chinese Medicine, Jinan, 250011 China; 2 Department of Gynecology and Obstetrics of Traditional Chinese Medicine, The First Clinical College, Shandong University of Traditional Chinese Medicine, Jinan, 250014 China; 3 Guangdong Provincial Key Laboratory of New Drug Screening, School of Pharmaceutical Sciences, Southern Medical University, Guangzhou, 510515 China

**Keywords:** Biological techniques, Diseases, Diseases, Biological techniques

## Abstract

The complexity of follicular fluid metabolome presents a significant challenge for qualitative and quantitative metabolite profiling, and for discovering the comprehensive biomarkers. In order to address this challenge, a novel SWATHtoMRM metabolomics method was used for providing broad coverage and excellent quantitative capability to discover the human follicular fluid metabolites related to recurrent spontaneous abortion (RSA) after *in vitro* fertilization and embryo transfer, and to evaluate their relationship with pregnancy outcome. The follicular fluid samples from the spontaneous abortion group (n = 22) and the control group (n = 22) were analyzed using ultra-performance liquid chromatography high-resolution mass spectrometry. A novel, high-coverage, targeted metabolomics method (SWATH to MRM) and a targeted metabolomics method were used to find and validate the differential metabolites between the two groups. A total of 18 follicular fluid metabolites, including amino acids, cholesterol, vitamins, fatty acids, cholic acid, lysophosphatidylcholine and other metabolites, were identified. In the RSA group, 8 metabolites, namely dehydroepiandrosterone, lysoPC(16:0), lysoPC(18:2), lysoPC(18:1), lysoPC(18:0), lysoPC(20:5), lysoPC(20:4), and lysoPC(20:3), were up-regulated, and 10 metabolites, namely phenylalanine, linoleate, oleic acid, docosahexaenoic acid, lithocholic acid, 25-hydroxyvitamin D3, hydroxycholesterol, 13-hydroxy-alpha-tocopherol, leucine, and tryptophan, were down-regulated. These differential metabolites related to RSA may provide a possible diagnostic basis and therapeutic target for RSA, as well as a scientific basis for elucidating the mechanism of RSA.

## Introduction

The occurrence rate of spontaneous abortion is approximately 10%, while the occurrence rate of recurrent spontaneous abortion (RSA) is around 5%^1^. Endocrine dysfunction^2–4^, anatomical factors^5–8^, genetic factors^9–11^, immunological factors^12,13^, infectious diseases^14^ and the age of the parents^15^ are considered as risk factors for spontaneous abortion. However, the causative factors for approximately 50% of RSA are still unknown, with the condition being termed unexplained RSA^16^. Since the “second child policy” was adopted in China in 2015, the proportion of older pregnant women has increased, and so has the number of RSA patients. Therefore, it is important and urgent to explore the unknown etiology of RSA. Follicular fluid (FF) provides the necessary micro-environment for oocyte growth, whereas the metabolites in FF can indirectly reflect the developmental potential of oocytes^17^. To date, there are no available studies on the relationship between oocyte quality and RSA.

Follicular fluid contains many complex components that have enormous structural diversity and a broad range of concentrations^18^. Therefore, a powerful analytical method with high sensitivity, broad coverage, specificity, and a wide dynamic range is required. High-resolution mass spectrometry, such as time-of-flight, was one of the most popular tools for analyzing complex metabolite profiling, including untargeted and targeted metabolomics^19,20^. Untargeted metabolomics using the sequential window acquisition of all theoretical fragment-ion spectra (SWATH) technique has a broad coverage in metabolite measurement but is limited by sensitivity, dynamic range, and reproducibility for complex biological samples^21^. Targeted metabolomics using multiple reaction monitor (MRM) technique was considered to be the gold standard for metabolite quantitation, as it is characterized by high sensitivity, a wide dynamic range, and good reproducibility; however, it is limited by low metabolite coverage^22^. Therefore, a novel SWATHtoMRM method was developed to acquire MS2 spectra of all precursor ions in a single analysis, and to extract a large-scale set of MRM transitions for targeted analysis with a high coverage in one experiment. Metabolomics profiling was performed in patients with RSA using the SWATHtoMRM method. We identified 18 RSA-associated metabolites, including cholesterol, vitamins, amino acids, fatty acids, cholic acid, and lysophosphatidylcholine, among others, that could provide a scientific basis for explaining the mechanism of RSA.

## Results

### Analytical characteristics of SWATHtoMRM method

Six compounds, namely d3-hexanoyl-carnitine, d5-L-tryptophan, d3-decanoyl-carnitine, PE (15:0/15:0), TG (15:0/15:0/15:0) and PC (17:0/17:0) were used as internal standards to assess the reliability of the SWATHtoMRM method. A series of concentrations of internal standards were prepared and added to the follicular sample. Six follicular fluid samples were prepared and analyzed in triplicate. The linear curve of each internal standard was constructed by its mean peak area at each concentration. The linear regression coefficients of d3-hexanoyl-carnitine, d5-L-tryptophan, d3-decanoyl-carnitine, PE(15:0/15:0), TG(15:0/15:0/15:0) and PC(17:0/17:0) were 0.9921, 0.9937, 0.9918, 0.9965, 0.9944 and 0.9953, respectively. The results revealed that the linear relationships were excellent.

Recovery was assessed at low, medium, and high concentrations for each internal standard. Before extraction or instrumental analysis, the mixture at each concentration was added to the follicular fluid matrix. The recovery experiment was carried out in three replicates of quality control (QC) samples. Recoveries were calculated by the peak area ratios of the standard spiked before extraction to the standard spiked before instrumental analysis. The results indicated that the recoveries ranged from 86.4% to 113.7% for six internal standards at low, medium, and high concentrations. Therefore, the recovery of our detection method was satisfactory.

The repeatability was evaluated by the relative standard deviation (RSD) of the ratios of the peak numbers and peak areas of six QC samples. For metabolomics analysis, 89.2% peaks occurred at RSD < 15% and accounted for 95.4% of the summed response in positive mode, while 92.7% peaks occurred at RSD < 15% and accounted for 93.9% of the total response in negative mode.

### Clinical background

In our present study, there were no statistically significant differences (P > 0.05) in patient age, infertility duration, BMI, Miscarriage history, gestational age of abortions, the level of bFSH, bLH and basal E2, the number of basal antral follicles (bAFC), the days of Gn and the dose of Gn, retrieved oocytes, metaphase II oocytes, fertilization rate and usable embryos. Therefore, the two groups were suitable for the comparative studies. The number of spontaneous abortions in the RSA group was 2.8 ± 0.55, compared to 0 in the control group (p < 0.001). The detailed results are shown in Table 1.

**Table 1:** The clinical background of control group and RSA group.

Clinical parameter	Control group	RSA group	T-test (*P*)
Age (years)	30.3 ± 3.5	29.7 ± 3.8	*0.696*
Infertility duration (years)	3.5 ± 1.9	3.1 ± 2.2	*0.338*
Body mass index (kg/m^2^)	21.7 ± 1.1	21.6 ± 1.2	*0.949*
Miscarriage history (years)	3.5 ± 1.3	3.2 ± 1.0	*0.395*
Number of spontaneous abortions (N)	0	2.8 ± 0.55	<*0.001*
Gestational age of abortion (weeks)	9.3 ± 2.3	9.8 ± 2.2	*0.465*
Basal FSH (mIU/mL)	6.8 ± 1.6	6.8 ± 1.9	*0.932*
Basal E2 (pg/mL)	46.8 ± 3.1	46.6 ± 3.7	*0.831*
Basal AFC (N)	15.8 ± 6.0	15.3 ± 5.1	*0.733*
Duration of Gn (days)	12.1 ± 2.4	12.1 ± 2.1	*0.953*
Dosage of Gn (mg)	2635.1 ± 987.3	2448.8 ± 828.1	*0.396*
Retrieved oocytes (n)	13.2 ± 3.5	12.2 ± 2.8	*0.301*
Metaphase II oocytes (n)	9.1 ± 2.3	8.9 ± 2.6	*0.788*
Fertilization rate (n, %)			*0.268*
IVF	13 (59.1%)	14 (63.6%)	
ICSI	9 (40.9%)	8 (36.4%)	
Usable embryos (n)	5.2 ± 2.8	4.5 ± 2.1	*0.354*

### Metabolites identification

**Figure 1:**
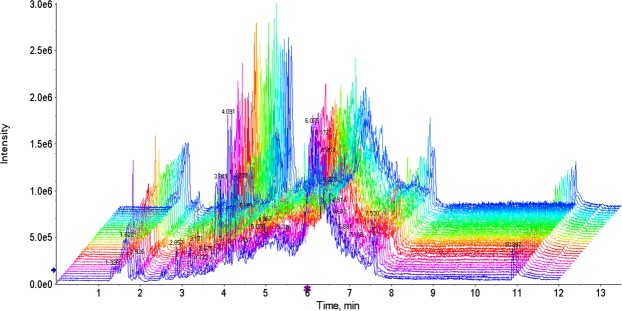
The total ion chromatography of all follicular fluid samples in positive mode.

**Figure 2:**
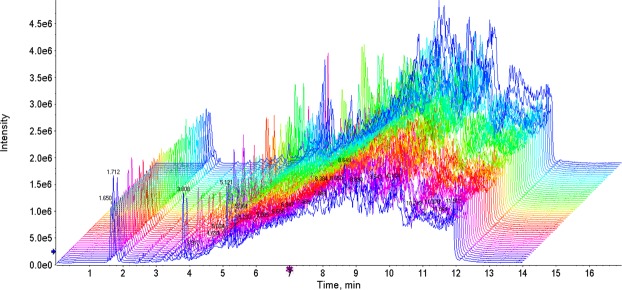
The total ion chromatography of all follicular fluid samples in negative mode.

**Figure 3:**
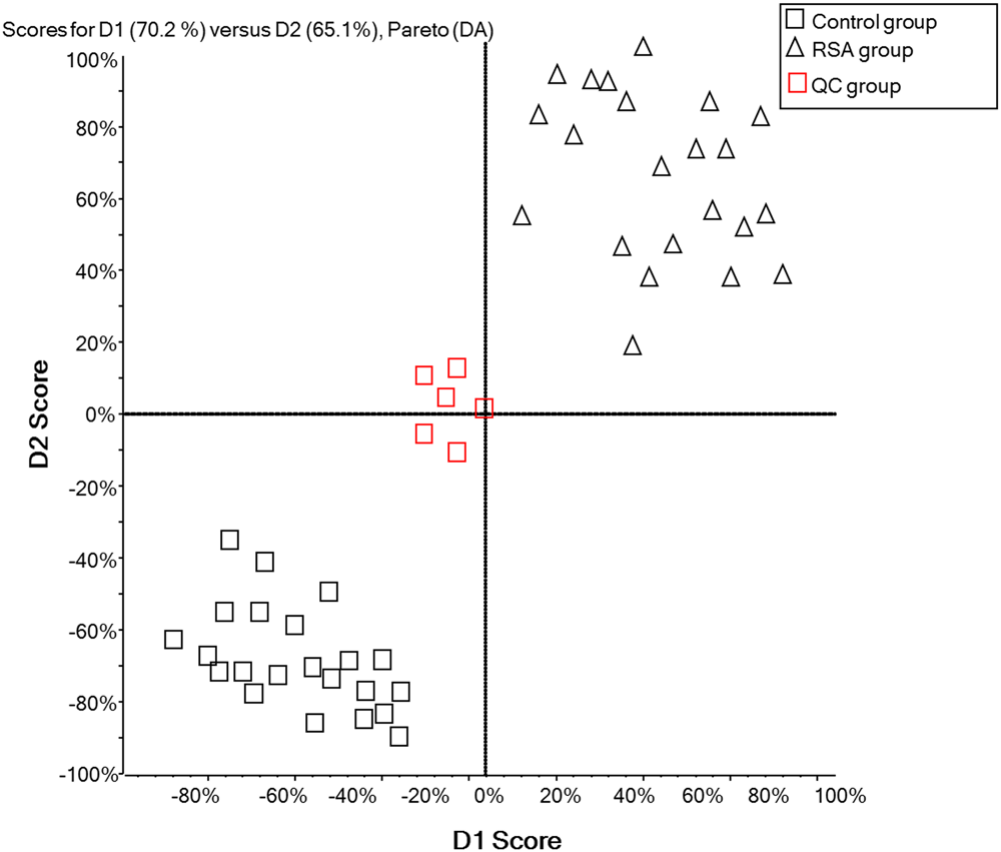
The score plot of PCA analysis of all FF samples in positive mode.

**Figure 4:**
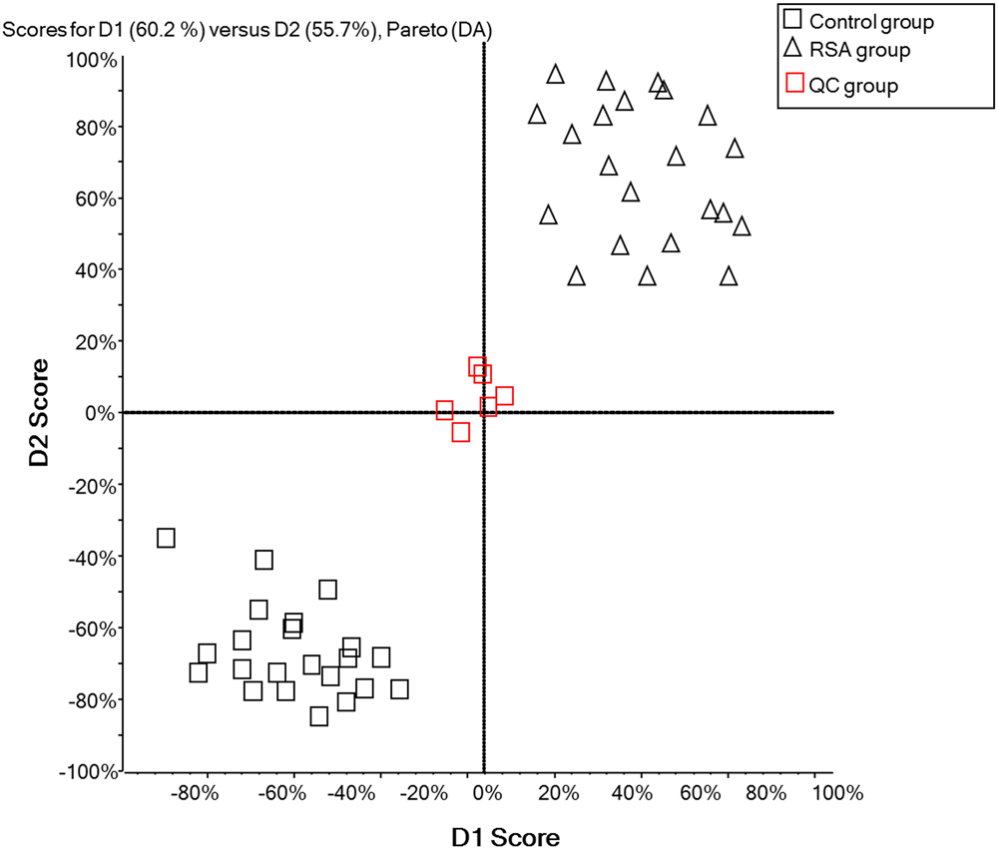
The score plot of PCA analysis of all FF samples in negative mode.

**Figure 5:**
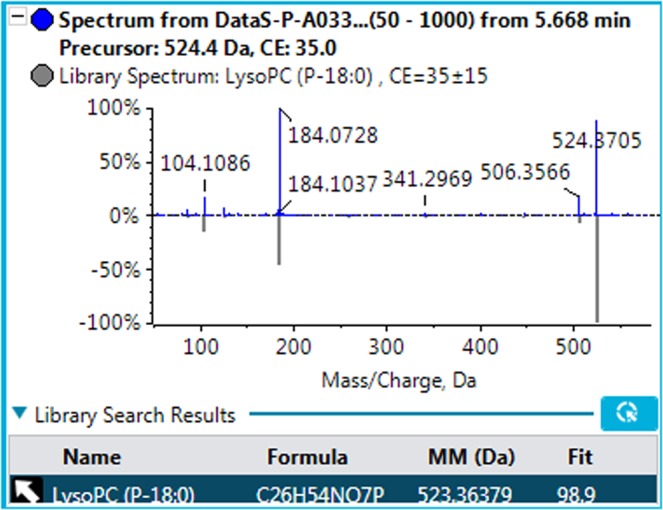
The MS2 spectra and matching result of M15.

The total ion chromatographs of all FF samples in positive mode and negative mode using UPLC-Q-TOF MS are shown in Figs 1 and 2, respectively. Thousands of compound features in follicular fluid were obtained. The QTOF data were converted to mzXML files using the “msconvert” program from ProteoWizard. Multiple data files were grouped and processed by SWATHtoMRM. A large-scale set of MRM transitions was produced, and a scheduled MRM method was performed. When the method is stable during sample analysis, QC FF samples will be tightly located in the score plot of the principal component analysis (PCA). As exhibited in Figs 3 and 4, QC FF samples were all tightly gathered in the PCA score in both positive and negative metabolomics analysis, indicating that the practicability of the method was good, and that the reproducibility of the analytical platform was excellent. The RSA group and control group were well-separated on the PC1 dimension, indicating that there are some changes in the FF metabolites of the SRA patients. The top 18 metabolites (VIP > 1 and p < 0.01) were considered as the potential differential metabolites. The differential metabolites were identified according to their accurate mass, isotope ratio, and MS/MS spectra. For example, differential metabolite M15 displayed the [M + H]^+^ ion at m/z 524.3710. The elution time of M15 was 6.1 min in the UPLC chromatogram. Its molecular formula was inferred as C_26_H_54_NPO_7_ according to its accurate mass and isotope patterns. A series of product ions were observed at m/z 506.3566, 184.0728, and 104.1086, as can be seen in Fig. 5. The fit of MS/MS matching reached greater than 95%. The structure of M15 was inferred as lysoPC(18:0).

**Figure 6:**
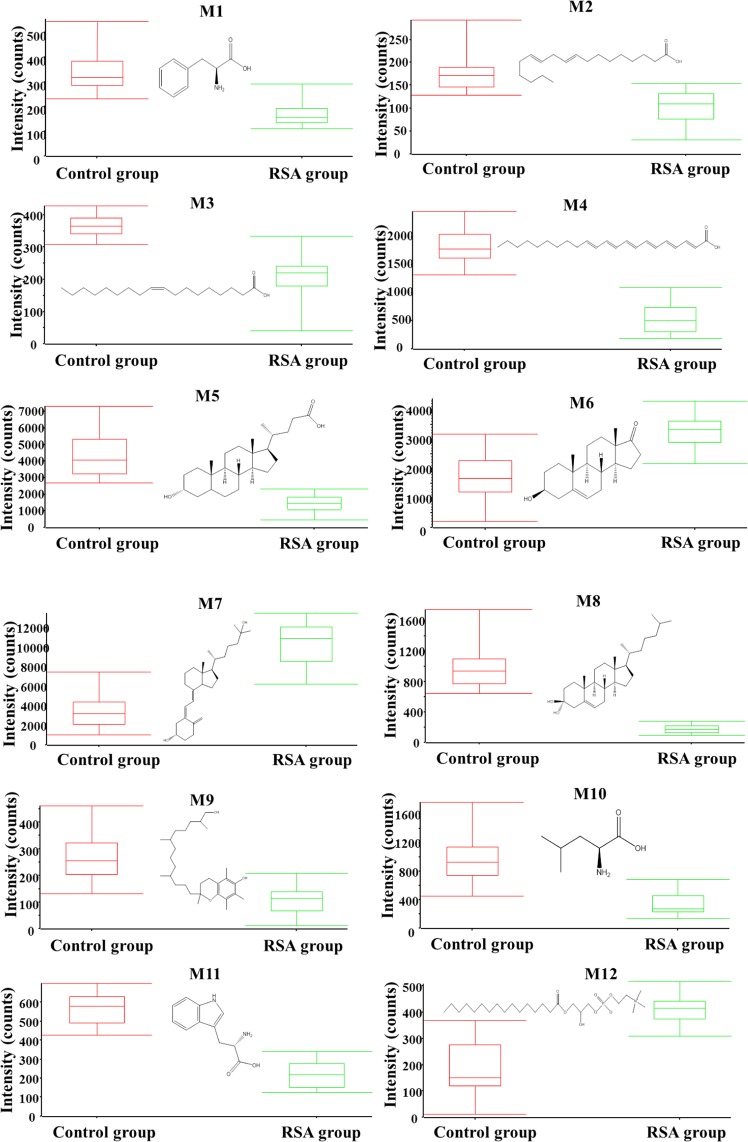
Metabolite profiles of the 12 candidate biomarkers (M1-M12) obtained from the quantitative analysis of the subjects (p < 0.05).

**Figure 7:**
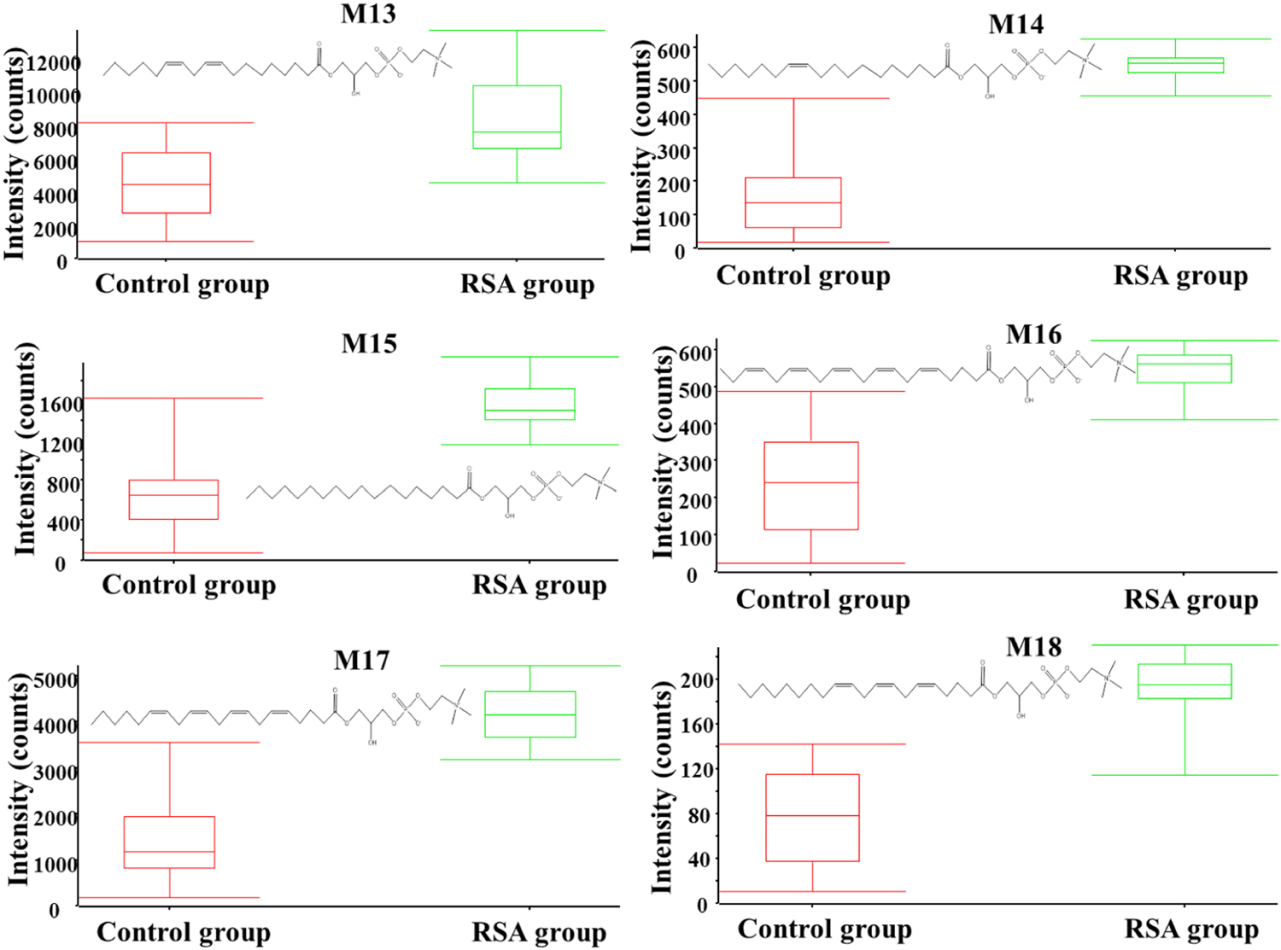
Metabolite profiles of the 6 candidate biomarkers (M13-M18) obtained from the quantitative analysis of the subjects (p < 0.05).

In total, 18 differential metabolites were identified as phenylalanine, linoleate, oleic acid, docosahexaenoic acid, lithocholic acid, dehydroepiandrosterone, 25-hydroxyvitamin D3, 25-hydroxycholesterol, 13-hydroxy-alpha-tocopherol, leucine, tryptophan, lysoPC(16:0), lysoPC(18:2), lysoPC(18:1), lysoPC(18:0), lysoPC(20:5), lysoPC(20:4) and lysoPC(20:3). These 18 standard compounds from Sigma-Aldrich were used to confirm their retention times and fragment ions. The detail information was shown in Table 2. In addition, the multiple reactions monitoring (MRM) transitions of the 18 potential metabolites (Table 3) were also targeted detected in all samples based on QTRAP5500. Statistical analysis was also conducted to validate the differences between the control group and the RSA group. The 18 differential metabolites were validated to be significantly different between the two groups. The final statistical results of screening method and targeted method are shown in Table 4. In the RSA group, 8 metabolites, namely dehydroepiandrosterone, lysoPC(16:0), lysoPC(18:2), lysoPC(18:1), lysoPC(18:0), lysoPC(20:5), lysoPC(20:4), and lysoPC(20:3), were up-regulated and 10 metabolites, namely phenylalanine, linoleate, oleic acid, docosahexaenoic acid, lithocholic acid, 25-hydroxyvitamin D3, hydroxycholesterol, 13-hydroxy-alpha-tocopherol, leucine, and tryptophan, were down-regulated. The differences in the 18 metabolites between the RSA group and the control group are displayed in Figs 6 and 7 with Graph Pad Prism. The structures of the metabolites are shown in the box plot. When accounting for outliers, the whiskers extend to a maximum of 1.5 times the inter-quartile range.

**Table 2:** Characterization of the biomarkers between control group and RSA group in follicular fluid.

Compound	T_R_ (min)	m/z	Polarity	Metabolites	Fragment ions (m/z)
M1	2.8	164.0718	−	Phenylalanine	72, 103, 147
M2	1.9	279.2310	−	Linoleate	197
M3	3.2	281.2487	−	Oleic acid	263
M4	8.6	327.2328	−	Docosahexaenoic acid	59, 135, 177, 229, 283
M5	10.0	375.2908	−	Lithocholic acid	59, 283
M6	5.1	287.2023	−	Dehydroepiandrosterone	97
M7	9.0	399.3266	−	25-Hydroxyvitamin D3	355, 271,
M8	9.6	401.3418	−	25-Hydroxycholesterol	321
M9	9.5	491.3715	−	13-hydroxy-alpha-tocopherol	445, 401
M10	2.6	132.1029	+	Leucine	69, 86
M11	3.2	205.0978	+	Tryptophan	118, 146, 188
M12	6.3	494.3246	−	LysoPC(16:0)	86, 184, 240, 339
M13	4.7	520.3394	+	LysoPC(18:2)	104, 184, 502
M14	5.2	522.3553	+	LysoPC(18:1)	104, 184, 504
M15	6.1	524.3710	+	LysoPC(18:0)	104, 184, 506
M16	4.9	542.3237	+	LysoPC(20:5)	104, 146, 337, 483
M17	5.6	544.3398	+	LysoPC(20:4)	104, 184, 485, 526
M18	6.6	546.3547	+	LysoPC(20:3)	104, 184, 341, 487

**Table 3:** The MRM parameters for targeted metabolomics method.

Compound	Q1/Q3	Dwell time (ms)	DP (V)	CE (eV)
Phenylalanine	164/147	10	−50	−15
	164/103	10	−50	−25
Linoleate	279/197	10	−50	−25
Oleic acid	281/177	10	−50	−30
	281/205	10	−50	−20
Docosahexaenoic acid	327/283	10	−50	−20
	327/229	10	−50	−35
Lithocholic acid	357/313	10	−50	−20
	357/259	10	−50	−28
Dehydroepiandrosterone	367/287	10	−50	−25
	367/97	10	−50	−40
25-Hydroxyvitamin D3	445/427	10	−50	−25
	445/401	10	−50	−35
25-Hydroxycholesterol	447/429	10	−50	−25
	447/403	10	−50	−30
13-hydroxy-alpha-tocopherol	491/473	10	−50	−19
	491/447	10	−50	−25
Leucine	132/86	10	50	18
	132/69	10	50	25
Tryptophan	205/188	10	50	20
	205/146	10	50	28
LysoPC(16:0)	494/476	10	−50	−20
	494/86	10	−50	−50
LysoPC(18:2)	520/184	10	50	35
	520/104	10	50	45
LysoPC(18:1)	522/184	10	50	35
	522/104	10	50	45
LysoPC(18:0)	524/184	10	50	35
	524 /104	10	50	45
LysoPC(20:5)	542/184	10	50	35
	542 /104	10	50	45
LysoPC(20:4)	544/184	10	50	35
	544/104	10	50	45
LysoPC(20:3)	546/184	10	50	35
	546/104	10	50	45

**Table 4:** Statistical results between control group and RSA group in follicular fluid.

Metabolites	Screening analysis			Targeted analysis		
	T-test (p)	VIP	FDR	T-test (p)	VIP	FDR
Phenylalanine	0.0076	2.13	0.48%	0.0009	1.89	0.08%
Linoleate	0.0012	2.78	0.12%	0.0028	1.44	0.02%
Oleic acid	0.0004	1.73	0.61%	0.0052	1.33	0.11%
Docosahexaenoic acid	0.0031	3.94	0.04%	0.0008	2.57	0.98%
Lithocholic acid	0.0073	2.21	0.21%	0.0081	2.16	0.32%
Dehydroepiandrosterone	0.0042	1.82	0.09%	0.0019	2.28	0.04%
25-Hydroxyvitamin D3	0.0061	1.76	0.43%	0.0017	1.46	0.33%
25-Hydroxycholesterol	0.0015	1.63	0.01%	0.0069	1.45	0.31%
13-hydroxy-alpha-tocopherol	0.0020	1.71	0.02%	0.0019	1.43	0.32%
Leucine	0.0001	2.11	0.07%	0.0022	1.62	0.15%
Tryptophan	0.0002	2.64	0.002%	0.0003	1.97	0.72%
LysoPC(16:0)	0.0011	2.87	0.08%	0.0073	2.03	0.08%
LysoPC(18:2)	0.0015	1.89	0.11%	0.0091	2.89	0.33%
LysoPC(18:1)	0.0081	1.68	0.29%	0.0068	1.47	0.09%
LysoPC(18:0)	0.0064	1.77	0.20%	0.0007	3.79	0.14%
LysoPC(20:5)	0.0047	1.86	0.41%	0.0023	1.72	0.02%
LysoPC(20:4)	0.0026	2.67	0.06%	0.0009	1.62	0.01%
LysoPC(20:3)	0.0041	2.31	0.04%	0.0012	1.63	0.09%

VIP: Variable important in the projection; FDR: false discovery rate.

### Pathway analysis

The related metabolic pathway analysis was performed on MetaboAnalyst. In the present study, abnormal changes were detected in aminoacyl-tRNA biosynthesis, phenylalanine, tyrosine, and tryptophan biosynthesis, nitrogen metabolism, linoleic acid metabolism, steroid hormone biosynthesis, and fatty acid biosynthesis.

## Discussion

Traditional untargeted metabolomics employs high-resolution MS, such as time-of-flight and Orbitrap, to detect as many metabolites as possible. However, the detector is readily saturated by high abundant ions in a full MS scan^23^, which makes the accurate measure of metabolites across a wide range of concentrations challenging. In comparison, traditional targeted metabolomics using the MRM technique, which has a good reproducibility, can precisely quantify a predefined set of known metabolites in biological samples^22^, but is primarily limited by low coverage. In our study, the SWATHtoMRM method, which is characterized by high coverage, high sensitivity, good reproducibility, and a wide dynamic range, was developed to measure the metabolites in follicular fluid.

The etiology of spontaneous abortion is complex, and includes chromosome genetic alterations, immune dysfunction, infections, male factors, uterine abnormalities, and environmental factors^24,25^. At present, the underlying cause of more than 50% of spontaneous abortions is still unknown. Oocytes quality may be closely related to the occurrence of spontaneous abortions. In our study, metabolomics based on the SWATHtoMRM method was employed to identify the related metabolites and metabolism pathway.

27-Hydroxycholesterol is a potent inhibitor of cholesterol synthesis, affecting the metabolism, transport and elimination of cholesterol. In a previous study, 27-hydroxycholesterol was shown to play a role in regulating cholesterol metabolism in early pregnancy. Cholesterol may affect trophoblast invasion and cause placental vasodilation and inflammation in early pregnancy. The imbalance of 27-hydroxycholesterol in early pregnancy could be a reason for the abnormal cholesterol metabolism. This, in turn, affects placental function resulting in miscarriage^26^.

Larkin found that 25-hydroxycholesterol could promote the release of human chorionic gonadotropin (hCG) and progesterone at a low concentration and inhibit the release of hCG and progesterone at a high concentration^27^. The levels of hCG and progesterone play an important role in maintaining pregnancy. In clinical practice, we found that low concentrations of 25-hydroxycholesterol affect the pregnancy outcome and result in miscarriage. Therefore, 25-hydroxycholesterol is closely related to spontaneous abortion.

Previous studies have shown that 25-hydroxyvitamin D3 has immunomodulatory effects^28–30^. Immune dysfunction is considered to be an important factor in RSA. Maternal systemic and peripheral immune regulation is crucial for fetal development. 25-hydroxyvitamin D3, as an immunomodulatory cytokine, could reduce the occurrence of RSA by preventing maternal rejection^31,32^. It could regulate the expression of homeobox A10 (key target gene of the implantation process) to improve endometrial receptivity and increase implantation rate^33^. Moreover, these results revealed high clinical pregnancy rates in IVF patients with high FF concentrations of 25-hydroxyvitamin D3. In our study, we found that the level of 25-hydroxyvitamin D3 was down-regulated in RSA group. Therefore, 25-hydroxyvitamin D3 plays an important role in the maintenance of pregnancy.

Simsek *et al*. found that lipid peroxidation is elevated in patients with RSA, while vitamin E, which is thought to reduce oxidative stress, is decreased^34^. This indicates that elevated oxidative stress and elevated lipid peroxidation are related to the occurrence of spontaneous abortions. 13′-hydroxy-alpha-tocopherol is a fat-soluble antioxidant, which can remove peroxyl radicals in the lipid oxidation process to protect polyunsaturated fatty acids against lipid peroxidation and reduce oxidative stress. Therefore, the lack of 13′-hydroxy-alpha-tocopherol may be a contributing factor of RSA. Miller *et al*. found that zebrafish with 13′-hydroxy-alpha-tocopherol deficiency can produce and lay eggs, however, after a few days, the resulting embryos suffered from stunting and death^35^. This indicates that 13′-hydroxy-alpha-tocopherol deficiency may be related to embryo death. In our study, the concentration of 13′-hydroxy-alpha-tocopherol was also found to be lower in RSA group. In another study, after the gene encoding 13′-hydroxy-alpha-tocopherol was knocked out in mice, abnormal embryonic development and embryo death was observed. Therefore, 13′-hydroxy-alpha-tocopherol plays a key role in embryonic development^36^.

Amino acids are very important for the proper growth and development of embryos. Certain amino acids, including tryptophan, leucine and phenylalanine, were found to be abnormal in the RSA group in our study. Trophoblast cells are essential cells in early pregnancy, supporting the development of both placenta and fetus. Functional defects of trophoblastic cells can lead to the reconstruction of spiral arteries and other gestational complications, such as RSA, intrauterine growth retardation, and preeclampsia. Zong *et al*. found that the expression and activity of indoleamine 2,3-dioxygenase (IDO) was low in patients with USRA^37^, which suggests that IDO could be closely related to RSA. This may be because IDO can inhibit the proliferation of local T cells and, thus, protect the embryo against an immune response^38^. In addition, tryptophan is metabolized to N-formyl kynurenine by IDO. In our study, the tryptophan metabolism pathway was abnormal in the RSA group, as seen in Table 2. This indicates that IDO was abnormal. Fei *et al*. also revealed that tryptophan metabolism and sphingolipid metabolism are important potential targets for miscarriage prevention, by comparing plasma metabolites between 33 patients with spontaneous abortion and 29 control subjects using UPLC^39^. Leucine and isoleucine belong to the branched-chain amino acids (BCAA). Zhang *et al*. found that BCAA are closely related to pregnancy outcome^40^. Elevated BCAA can cause low pregnancy rates and high the abortion rates. In a different study, Van *et al*. showed that leucine, isoleucine and methionine are closely related to embryo development^41^. A previous study found that hyperphenylalaninemia has adverse effects on early fetal development. It can cause spontaneous abortion, fetal growth retardation and skeletal deformities^42^. Banerjee *et al*. found that lysine, L-arginine, glutamine, threonine, histidine, phenylalanine and tyrosine were significantly increased in patients with RSA. These altered metabolites may be involved in excessive inflammatory reactions and vascular dysfunction related to poor endometrial receptivity^43^. Leucine is also closely related to embryonic development. Research has shown that the level of serum leucine aminopeptidase is very low in fetal death patients^44^. Therefore, serum leucine aminopeptidase could be an effective predictor of fetal death. Pogorelova also found that the levels of leucine, threonine and tyrosine were low in patients with fetal growth retardation. Therefore, low levels of leucine have a negative effect on the growth and development of the embryo, causing growth retardation or stillbirths^45^.

Polyunsaturated fatty acids (PUFA) play an important role in oocyte maturation and embryonal development. It was found that docosahexenoic acid (DHA) can improve the quality of oocytes and increase the blastocyst rate of bovine oocytes^46^. Therefore, DHA has a positive effect on oocyte and embryo quality. Monounsaturated fatty acids (MUFA) such as oleic acid play an important role in oocyte maturation and embryonal development. High intake of MUFA can increase the live birth rate after embryo transfer^47^. Palmitic acid and stearic acid could inhibit the proliferation of granulosa and theca cells and induce cell apoptosis, whereas oleic acid could reduce these negative effects^48^. Some researchers believe that a high level of linoleic acid negatively impacts oocyte maturation and development^49^. It can also affect the human reproductive system and reduce the pregnancy rate after *in vitro* fertilization. Therefore, oleic acid and linoleic acid are related to oocyte maturation and embryonal development. Colvin *et al*. found that palmitate can increase the death of syncytial trophoblasts and the pressure on the endoplasmic reticulum^50^. The toxicity of palmitate on the human syncytial trophoblasts could cause spontaneous abortion by affecting embryonic development.

Chen *et al*. found that total bile acids are a valuable indicator of adverse outcomes of the perinatal period. Mono hydroxy bile acids have toxic effects on the hepatobiliary system of the fetus and newborn^51^. Siviero *et al*. found that cholic acid and lithocholic acid could lead to miscarriage due to inflammation and hepatocyte degeneration^52^.

Li showed that lysophosphatidylcholine (LysoPC) may be related to adverse pregnancy outcomes^53^. Chen also proved that LysoPC is closely related to the occurrence of embryo arrest^54^. In our study, LysoPC(16:0), LysoPC(18:3), LysoPC(18:2), LysoPC(18:1), LysoPC(18:0), LysoPC(20:5), LysoPC(20:4) and LysoPC(20:3) were upregulated in RSA group. These results indicate that lipids metabolism was abnormal.

Dehydroepiandrosterone and dehydroepiandrosterone sulfate (DHEAS) are mainly produced in the adrenal gland^55^. Leigh *et al*. found that the level of DHEAS is high in patients with RSA^56^, which indicates that DHEAS could be a determinant of spontaneous abortion. In our study, we also found that DHEAS was higher in patients with spontaneous abortion. Dendritic cells (DC) are a key regulator of immune tolerance during pregnancy. The functional impairment of DC has been considered as one of the pathologic factors of RSA^57^. Chernykh *et al*. showed that DC impairment was found in patients with elevated DHEAS^58^. High levels of DHEAS could cause spontaneous abortions by causing abnormal immunization in pregnant women. In addition, we found that the level of linoleate in the RSA group were also downregulated. However, their relationship to spontaneous abortion is still unknown.

In this study, the advanced SWATHtoMRM method, which has high coverage and high sensitivity, was used for exploring the differential metabolites between the RSA group and the control group. Dehydroepiandrosterone, lysoPC(16:0), lysoPC(18:3), lysoPC(18:2), lysoPC(18:1), lysoPC(18:0), lysoPC(20:5), lysoPC(20:4), lysoPC(20:3), phenylalanine, linoleate, oleic acid, docosahexaenoic acid, lithocholic acid, 25-hydroxyvitamin D3, 25-hydroxycholesterol, 13-hydroxy-alpha-tocopherol, leucine and tryptophan were identified.

There are some potential limitations of this study that must be considered when interpreting our conclusions. First, the number of patients in this study was relatively small. Second, it is indispensable that, in further studies, a larger number of subjects with RSA should be obtained in prospective validation experiments to verify the present results. Third, follicular fluid can merely be obtained from patients performing oocyte retrieval. Therefore, our findings can only provide indirect evidence for the pathogenesis of RSA.

This study suggests that FF metabolic profiling has great potential in differentiating RSA patients from control patients undergoing IVF treatment, implying that the differential metabolites might be novel biomarkers of RSA, and can be utilized to identify RSA before it occurs so that appropriate measures can be taken in the future. These differential metabolites of FF related to RSA are expected to provide an improved understanding of the disease pathogenesis.

## Methods

### Subjects

For the metabolomics analysis, the MetSizeR approach for sample size estimation was used to estimate a total sample size of 44 subjects using the following assumptions: spectra of 584 spectral bins, a target false detection rate of 5%, and an expected proportion of significant spectral bins of 20%^59^. Subjects needed (n = 44) were recruited and their FFs was collected at the affiliated hospital of Shandong University of Traditional Chinese Medicine, from May 2016 to February 2017. For the purpose of this study, the subjects were divided into the RSA group (n = 22) and the control group (n = 22). The study was approved by the Health Authorities and Ethics Committees of the Affiliated Hospital of Shandong University of Traditional Chinese Medicine. All subjects signed the informed consent prior to being included in the study.

### Inclusion and exclusion criteria

Inclusion criteria: (1) all patients received IVF treatment due to fallopian tube factors; (2) patients of the RSA group who had experienced more than 2 unexplained and consecutive spontaneous abortions at less than 10 weeks of gestation; (3) patients of the control group who had a history of induced abortions of a normal pregnancy.

Exclusion criteria: (1) those who had genital abnormalities, chronic hypertension, diabetes, autoimmune diseases, infectious diseases, or liver, kidney, cardiovascular, or thyroid diseases; (2) the subjects with body mass index (BMI) values greater than 30 kg/m^2^ and age ≥40 years old.

### Sample collection

GnRH-a was used for ovulation induction in both the RSA group and the control group. During the previous menstrual cycle, 0.05 mg of Diphereline (Decapeptyl®, Ipsen Pharma Biotech) was injected at the beginning of the medium-term corpus luteum. As the pituitary-regulated hormone secretion was suppressed (maximum follicle diameter ≤0.7 cm, endometrial thickness ≤5 mm; level of follicle stimulating hormone (FSH) and luteinizing hormone (LH) < 10 U/L; level of estradiol (E2) < 146 pmol/L), the recombinant FSH was triggered. Follicles larger than 18 mm in diameter were aspirated in 36 hours. After oocyte isolation, FF was pooled and centrifuged at 14,000 × g for 20 min to remove cells and insoluble particles. The supernatant was then transferred to sterile cryovials and stored at −80 °C for further study. All operations were performed in accordance with ISO 9001:2008. Subsequently, an elective freeze-all strategy was performed; all embryos were vitrified as cleavage stage embryos on Day 3.

### Sample preparation

Follicular fluid samples of 200 μL were mixed with 600 μL of methanol/isopropanol/water (4:4:2) containing six internal standards: d3-hexanoyl-carnitine, L-tryptophan-d5, d3-decanoyl-carnitine, PE (15:0/15:0), TG (15:0/15:0/15:0) and PC (17:0/17:0). The mixture was vortexed for 5 min and then centrifuged at 14000 × g for 30 min, at 4°C. The supernatant was then transferred to an autosampler plate for analysis.

### LC-MS condition

A SCIEX ExionLC AD ultra-performance liquid chromatography (UPLC) system and a reverse-phase ACQUITY UPLC® BEH C18 column (2.1 × 100 mm, 1.7 μm) were used for metabolomics analysis. The follicular fluid of 5 μL was injected at 15 °C. The total flow rate was set at 0.4 mL/min. The column temperature was set at 40 °C. In positive mode, water with 0.1% formic acid (FA) was used as mobile phase A and acetonitrile with 0.1% FA was used as mobile phase B. The elution gradient was kept at 95% A for 0.5 min, increased to 100% B over the next 7 min, and then returned to 95% A from 10 min to 10.1 min. The total run time was 12 min. In negative mode, water with 5 mM of ammonium acetate was used as mobile phase A, and acetonitrile was used as mobile phase B. The elution gradient was kept at 95% A for 0.5 min, increased to 100% B over the next 8 min, and then returned to 95% A from 12 min to 12.1 min. The total run time was 14 min. All SWATH data were acquired on a SCIEX Triple TOF 5600^+^, and all MRM data were acquired on a SCIEX QTRAP 5500. The nebulizer gas (GS1 and GS2) was set at 55 psi. The source temperature was set at 550 °C. In positive mode, the voltage of ion spray was 5,500 V. The declustering potential and collision energy were set at 60 V and 35 ± 15 V, respectively. In negative mode, the voltage of ion spray was −4,500 V. The declustering potential and collision energy were set at −60 V and −35 ± 15 V, respectively. The full scan range and the product ion scan range were all from m/z 50 to m/z 1200. The raw SWATH data were converted to mzXML files using the “msconvert” program from ProteoWizard. Multiple data files were grouped and processed by SWATHtoMRM. A large-scale set of MRM transitions was produced, and a scheduled MRM method was then constructed using Analyst TF 1.7.1 software to maximize the number of measured MRM transitions in each analysis.

### Data processing and statistical analysis

In total, 44 follicular fluid samples were analyzed in replicates using UPLC-TOF and UPLC-QTRAP. Data were processed using the PeakView software and the MarkerView software for peak detection, the extraction of MS2 peaks and chromatograms, and MS1 and MS2 peak grouping. According to the “80% rule”, peaks present in more than 80% of the samples of each group were kept for further analysis. In large-scale metabolomics measurements, the reproducibility of the analysis may be influenced by source contamination or the maintenance and cleaning of the mass-spectrometer. Normalization is a common preprocessing method to decrease systematic change. In our study, peak areas of all metabolites were normalized. Only the metabolites with an RSD value below 15% in QC samples were used for statistical analysis. Principal component analysis (PCA) was used to discover differential variation features on the MarkerView software. Univariate statistical analysis was performed by using the Student’s t-test. Variables with univariate statistical significance (p < 0.05) were considered markedly different between the two groups. In addition, supervised orthogonal partial least-squares discriminant analysis (OPLS-DA) was also applied to model all features of the two groups in MetaboAnalyst (http://www.metaboanalyst.ca). The predictability of the model was determined by internal validation with 7-fold cross-validation and response permutation testing. Variable importance in the projection (VIP) value of all variables was explored from the best-fitted OPLS-DA models. Significance analysis of microarray (SAM) was also performed to address the false discovery rate (FDR) for multiple tests. Differential variables with a VIP value greater than 1 and an FDR value less than 0.05 were selected. Therefore, potential differential variables were validated by p value, VIP value and FDR value. These differential variables were identified by accurate mass, isotope patterns, and mass spectrometric fragmentation patterns, which were then used to search databases, including KEGG, PubChem compound, METLIN, the Madison Metabolomics Consortium Database and the Human Database.

### Targeted metabolomics study

Targeted metabolomics analysis was also carried out using an UHPLC system (LC-30AD, Shimadzu) coupled to a Turbo V electrospray ionization source and a Qtrap 5500 mass spectrometer. The MS detection was performed using MRM transitions, according to Table 3. All 18 metabolites were targeted in a single injection using both positive and negative modes with rapid polarity switching (50 ms). The data were processed in MultiQuant 3.0 (SCIEX). Statistical analysis was also performed in MetaboAnalyst.

### Ethical considerations

All experiments were performed in accordance with institutional guidelines and approved by the Health Authorities and Ethics Committees of the Affiliated Hospital of Shandong University of Traditional Chinese Medicine. All subjects signed the informed consent prior to being included in the study.

## Data Availability

The datasets generated during the present study are available from the corresponding author on reasonable request.
